# Evaluation of an alpha-cypermethrin + PBO mixture long-lasting insecticidal net VEERALIN® LN against pyrethroid resistant *Anopheles gambiae* s.s.: an experimental hut trial in M’bé, central Côte d’Ivoire

**DOI:** 10.1186/s13071-019-3796-x

**Published:** 2019-11-15

**Authors:** Welbeck A. Oumbouke, Mark Rowland, Alphonsine A. Koffi, Ludovic P. A. Alou, Soromane Camara, Raphael N’Guessan

**Affiliations:** 1 London School of Hygiene and Tropical Medicine, Keppel Street, London, UK; 2 Vector Control Product Evaluation Centre (VCPEC)/Institut Pierre Richet, 01 BP 1500, Bouaké, Côte d’Ivoire

**Keywords:** *Anopheles gambiae* s.s., Experimental hut, Insecticide resistance, PBO, Long-lasting insecticidal net

## Abstract

**Background:**

Long-lasting insecticidal nets (LLINs) are the primary method of malaria prevention. However, the widespread resistance to pyrethroids among major malaria vector species represents a significant threat to the continued efficacy of pyrethroid LLIN. Piperonyl butoxide (PBO) is a synergist that inhibits the activity of metabolic enzymes of the cytochrome P450 family known to detoxify insecticides including pyrethroids. Synergist LLIN incorporating PBO and a pyrethroid may provide improved control compared to pyrethroid-only LLIN.

**Methods:**

The efficacy of VEERALIN® LN (VKA polymers Pvt Ltd, India), an alpha-cypermethrin PBO synergist net was evaluated in experimental huts in M’bé, central Côte d’Ivoire against wild pyrethroid resistant *Anopheles gambiae* s.s. Comparison was made with a standard alpha-cypermethrin-treated net (MAGNet® LN, VKA polymers Pvt Ltd, India). Nets were tested unwashed and after 20 standardized washes.

**Results:**

VEERALIN® LN demonstrated improved efficacy compared to MAGNet® LN against wild free-flying pyrethroid-resistant *An. gambiae* s.s. Before washing, VEERALIN® LN produced mortality of *An. gambiae* s.s. (51%) significantly higher than the standard pyrethroid-only net (29%) (*P* < 0.0001). Although there was a significant reduction in mortality with both LLINs after 20 washes, VEERALIN® LN remained superior in efficacy to MAGNet® LN (38 *vs* 17%) (*P* < 0.0001). Blood-feeding was significantly inhibited with both types of insecticide-treated nets relative to the untreated control net (*P* < 0.0001). Unwashed VEERALIN® LN induced significantly higher blood-feeding inhibition of *An. gambiae* s.s. (62.6%) compared to MAGNet® LN (35.4%) (*P* < 0.001). The difference persisted after washing, as there was no indication that either LLIN lost protection against biting or blood-feeding. The level of personal protection derived from the use of VEERALIN® LN was high (87%) compared to MAGNet® LN (66–69%) whether unwashed or washed. The AI content of VEERALIN® LN after 20 washes decreased from 6.75 to 6.03 g/kg for alpha-cypermethrin and from 2.95 to 2.64 g/kg for PBO, corresponding to an overall retention of 89% for each compound.

**Conclusions:**

The addition of the synergist PBO to pyrethroid net greatly improved protection and control of pyrethroid-resistant *An. gambiae* s.s. The pyrethroid-PBO VEERALIN® LN has the potential to reduce transmission in areas compromised by pyrethroid resistance.

## Background

Long-lasting insecticidal nets (LLINs) are considered best practice for malaria prevention in the majority of African countries. The estimated proportion of people sleeping under nets in sub-Saharan Africa rose to 53% in 2015 from a low of less than 2% in 2000. This increase in net use has resulted in about half a billion clinical malaria cases averted over the same time period [1]. This substantial reduction in malaria cases justifies ongoing efforts by National Malaria Control Programmes (NMCPs) to increase ownership and use of LLIN.

Despite the significant headway made, malaria remains a major public health problem in many countries. Recent estimates from the WHO World Malaria Report indicate that progress has stalled between 2015 and 2017, with some countries even reporting an increase in the number of cases [2]. One potential factor contributing to this is the rise in resistance to pyrethroid insecticides in malaria vectors across Africa. Although some malaria and health facility surveys in Benin have not provided evidence that resistance is adversely affecting malaria transmission or burden [3, 4], household and hut trials [5, 6] indicate that pyrethroid resistance can significantly reduce the efficacy of standard LLIN for vector control and personal protection. While findings from these malaria and health facility surveys in Benin suggested no association between pyrethroid resistance and malaria transmission, these are observational studies and therefore provide no conclusive evidence on the impact of resistance. Moreover, malaria prevalence remains high in many areas of Benin despite the widespread use of LLIN. This emphasises the need for additional control measures to improve control and reduce malaria transmission.

Although LLIN may provide some protection against insecticide-resistant *Anopheles* mosquitoes, this may depend on the frequency and strength of the resistance [7–9]. To meet the resistance challenge and restore malaria vector control, new active ingredients are being developed and tested. A new class of net combines two compounds: the pyrethroid and the synergist piperonyl butoxide (PBO) for improved control of pyrethroid-resistant anopheline mosquitoes. PBO is an insecticide synergist which inhibits the action of resistance-associated metabolic enzymes of the cytochrome P450 family [10]. The inhibition of P450 enzymes by the PBO results in the pyrethroid on the net being available to induce excito-repellency and mortality. The role of these enzymes in the detoxification of insecticides including pyrethroids and to cause resistance is well documented [11–14]. The addition of PBO to pyrethroid net as a strategy to overcome resistance especially in areas where this is driven by overexpression of P450 enzymes known to metabolise pyrethroids has been demonstrated in a range of experimental hut trials across Africa [15–19]. Simulation modelling suggests that a switch in net policy toward pyrethroid-PBO net would result in up to 0.5 clinical malaria cases averted per 1000 people per year [20]. Pyrethroid-PBO net was given World Health Organisation (WHO) policy recommendation as a new class in 2017 based on epidemiological data from a cluster randomized trial in Muleba, Tanzania [21], which showed that Olyset® Plus LN (Sumitomo Chemicals Co. Ltd, Tokyo, Japan) reduced malaria-infection prevalence by 33% over 21 months of use compared to the standard LLIN, Olyset® Net, under a scenario of high pyrethroid resistance and net use. A recent Cochrane review predicted that PBO-pyrethroid LLIN is expected to be more effective in areas of moderate to high resistance mediated by metabolic resistance than in settings of low or no insecticide resistance [22].

The recommendation of new product class applies to all pyrethroid-PBO nets prequalified by the WHO [23]. All of these products differ from Olyset® Plus in terms of their design/specifications, which in turn is likely to affect their field performance. Key differences between these products include the spatial location of the PBO (all net panels or just the top panel), PBO loading dose, type and concentration of pyrethroid and wash-fastness and bioavailability of PBO or partner pyrethroid. VEERALIN® LN (VKA polymers Ltd, Tamil Nadu, India) is a new PBO-alphacypermethrin synergist LLIN that contains PBO on all net panels and recently acquired WHO interim recommendation. The Vector Control Product Evaluation Centre (VCPEC) based within Institut Pierre Richet (IPR) in Bouaké, central Côte d’Ivoire was therefore commissioned by the WHO to undertake a phase-2 experimental hut study of VEERALIN® LN in an area of high pyrethroid resistance mostly mediated by cytochrome P450 metabolic mechanisms.

## Methods

### Study area and experimental huts

The hut trial was conducted at the M’bé field station in central Côte d’Ivoire, 40 km south of Bouaké city. The site is a large rice irrigated valley producing mostly *An. coluzzii* year round. The mosquito population from the site has developed resistance to multiple insecticide classes. Resistance mechanisms include target site insensitivity (1014F and *Ace-1*) [24] and increased activities of insecticide-metabolizing enzymes (esterases, oxidases and GSTs) [25] including highly overexpressed CYP6P3 (Oumbouke and N’Guessan, in preparation). A recent investigation into the level of resistance to pyrethroids in *Anopheles-*mosquitoes from the study area reported over 1700-fold resistance to deltamethrin [26].

The West African style experimental huts were used for the field trial [27]. They were made of concrete bricks, with roofs of corrugated iron, ceilings lined with plastic sheeting and the interior walls plastered with cement. Each hut was built on a concrete base surrounded by a water-filled moat to prevent entry of mosquito predators. Mosquitoes enter the hut through four 1-cm wide window slits, located on three sides of the hut. Mosquitoes exiting the hut are caught in a veranda trap located on the fourth side.

### WHO susceptibility assays

To determine the prevalence of resistance to pyrethroids, WHO cylinder assays were conducted using papers treated with diagnostic concentration of 0.05% alpha-cypermethrin, the same pyrethroid used in MAGNet® and VEERALIN® LLINs. WHO susceptibility tests were performed using 2–3 day-old adult female mosquitoes, collected as larvae from the M’bé field station. Four replicates of 25 female mosquitoes were tested in cylinder assays and mortality was scored 24 h after exposure. Exposure of the susceptible *An. gambiae* strain to alpha-cypermethrin treated paper in cylinder tube was conducted to check the quality of the insecticide-treated paper. Mosquitoes exposed to untreated paper served as control.

### LLINs and washing procedure

MAGNet® LN is a long-lasting net containing 5.8 g/kg alpha-cypermethrin incorporated in monofilament, high-density polyethylene (HDPE), 150-denier manufactured by VKA polymers. MAGNet® LN received full WHOPES recommendation in 2011 [28].

VEERALIN® LN is a long-lasting net manufactured by VKA polymers Pvt Ltd, India. Alpha-cypermethrin is incorporated into 130-denier monofilament polyethylene fibres with a target dose of 6.0 g AI/kg (216 mg AI/m^2^) alpha-cypermethrin and 2.2 g/kg (79.2 mg/m^2^) PBO.

The nets were washed following the WHOPES-phase II washing protocol [29]. The time for regeneration of the active ingredients between washes was 1 day for MAGNet® LN and 5 days for VEERALIN® LN and therefore washing was done every 5 days using 2 g/l soap solution (‘savon de Marseille’). One complete washing cycle of each net ran for 10 min as follows: each net was first agitated for 3 min then left to soak for 4 min and again agitated for 3 min. Net agitation was performed by stirring each net with a wooden pole at 20× *rpm*. After washing, nets were rinsed twice in clean water (10 l per rinsing, i.e. 20 l per net). Nets were dried horizontally in the shade, then stored at ambient temperature (27 ± 2 °C) between washes.

### Net treatments and experimental hut trial procedure

The following treatment arms were trialed in experimental huts: (i) VEERALIN® LN unwashed; (ii) VEERALIN® LN washed 20 times; (iii) MAGNet® LN unwashed; (iv) MAGNet® LN washed 20 times; and (v) untreated polyester net (100 denier).

These five treatment arms were randomly allocated to 5 experimental huts. To account for potential bias due to differential hut attractiveness, nets were rotated among huts every week according to a balanced Latin square scheme. Three nets were used per treatment arm and each net was tested within hut on 2 consecutive nights during the week. Before the hut trial, holes (16-cm^2^ in diameter) were made in the nets (2 on each side and 1 on each end) to simulate moderately damaged net during field use. The huts were thoroughly cleaned and aired for a day at the end of each rotation.

The hut trial spanned 5 weeks (from June to July 2014) corresponding to 30 nights of collection per hut. Five local human volunteers gave informed consent and slept in the huts from 20:00 h to 05:00 h each night. To reduce bias resulting from the inherent difference in individual attractiveness to host-seeking mosquitoes, sleepers were rotated between huts on successive nights. Each morning, mosquitoes were collected from huts using mouth-operated aspirators from inside the room, nets and veranda traps and physiological status (live, dead, unfed, blood-fed, semi-gravid, gravid) recorded. Mosquitoes were transported to the laboratory at the Institut Pierre Richet (IPR), Bouaké, Côte d’Ivoire, and identified to the species level. Live female mosquitoes were provided with 10% honey solution and mortality recorded 24 h later.

### Outcome measures

The following outcomes were used to assess the efficacy of the treatments as per WHO guidelines [30]: (i) deterrence: the percent reduction in the number of mosquitoes in treatment hut relative to control hut with untreated net; (ii) exit rate; (iii) blood-feeding inhibition rate: the percentage reduction in blood-feeding in a hut with treated net compared to a hut with untreated net; (iv) percentage mortality of adult females; (v) overall insecticidal effect = 100 (Kt − Ku)/Tu, where Kt is the number of mosquitoes killed in the treated hut, Ku is the number dying in the untreated control hut and Tu is the total number collected from the control hut [5]; (vi) personal protection; percentage reduction in mosquito biting in hut with treated net compared to hut with untreated net = [1 − (No. of mosquitoes blood-fed in treatment/No. of mosquitoes blood-fed in control) × 100].

### Chemical analysis

Determination of alpha-cypermethrin content in unwashed and washed MAGNet® and VEERALIN® LLINs was performed before and after washing and post-trial in accordance with WHO guidelines [29]. PBO content was also assessed in VEERALIN® LN. A piece of netting measuring 30 cm × 30 cm was cut from each of the five locations of each net. Extraction of alpha-cypermethrin and PBO was performed using the CIPAC method [31]. These compounds were extracted by refluxing with xylene for 30 min in the presence of dioctyl-phthalate as an internal standard and citric acid. Concentrations of alpha-cypermethrin and PBO were subsequently measured by Gas Chromatography with Flame Ionization Detection (GC-FID).

### Cone bioassays

The efficacy of VEERALIN® and MAGNet® LLINs was assessed by WHO cone bioassay using susceptible *An. gambiae* before and after washing and after field trial. Hundred 2–5 day-old female mosquitoes were subjected to 3 min exposure in replicates of 5 mosquitoes per cone at 25 ± 2 °C and a relative humidity of 75 ± 10% [30]. Mortality was scored 24 h after exposure.

### Statistical analysis

Data were entered into an Excel database and subsequently imported into the R statistical software version 2.15.0. for analysis. Proportional outcomes from the bioassays (mortality) and the hut trial (exophily, blood-feeding and mortality) were analysed using generalised linear mixed models (GLMMs) with a binomial distribution and a logit link function was fitted to the data using the *lme4* package [32]. Net type and hut were included as fixed effects, and sleepers and day of mosquito collection were treated as random effects. Numeric outcomes (number entering each hut, feeding and dying) were analysed using generalised linear models with a Poisson distribution. Pairwise comparisons were performed using the *multcomp* package in R [33].

## Results

### WHO susceptibility assays

Mortality of the susceptible *An. gambiae* exposed to 0.05% alpha-cypermethrin in WHO susceptibility tests was 100%. Mortality of *An. gambiae* s.s. from M’bé exposed to the diagnostic dose of alpha-cypermethrin was 68% (*n* = 108), indicating frequency of resistance to pyrethroids of 32% at the study site.

### Experimental hut trial

In the 5-week trial, 1054 *An. gambiae-*mosquitoes were collected from the control hut, representing a mean number of 29 females per night. Both MAGNet® and VEERALIN® LLINs reduced hut entry of *An. gambiae* s.s.; unwashed MAGNet® LN reduced entry by 52% and unwashed VEERALIN® LN by 65%. There was no evidence of reduced entry after washing the two LLINs 20 times (Table 1, Additional file 1: Table S1, Additional file 2: Table S2).

**Table 1:** Experimental hut trial results of unwashed and 20-times washed pyrethroid-PBO and pyrethroid-only LLIN against pyrethroid resistant *Anopheles gambiae* s.s. in M’bé, Côte d’Ivoire

	Untreated net	MAGNet® LN 0w	MAGNet® LN 20w	VEERALIN® LN 0w	VEERALIN® LN 20w
Total no. of females caught	1054	506	519	366	377
Mean no. caught/night	29.2^a^	14.0^b^	14.4^b^	10.2^c^	10.5^c^
% Deterrence	–	52.0	50.7	65.3	64.2
Total no. of females in veranda	248	279	243	203	244
% Exiting (95% CI)	23.5 (21.0–26.1)^a^	55.1 (50.8–59.5)^b^	46.8 (42.5–51.1)^c^	55.5 (50.4–60.6)^b^	64.7 (59.9–69.5)^d^
Total no. of blood-fed females	665	206	225	86	89
% Blood-feeding inhibition	–	35.5 (31.3–39.7)	31.4 (27.4–35.4)	62.7 (57.7–67.6)	62.6 (57.7–67.5)
% Personal protection	–	69.0^a^	66.2^a^	87.1^b^	86.6^b^
Overall insecticidal effect (%)	–	11.8^a^	6.4^b^	15.5^a^	11.5^a^

*Note*: Values in the same row sharing a letter superscript do not differ significantly (*P* > 0.05, GLMMs)

Relative to the untreated control, the proportions of mosquitoes exiting into the verandas was significantly greater with each type of insecticide treated net by 47–65% (GLMMs, *P* < 0.0001) (Table 1, Additional file 1: Table S1, Additional file 2: Table S2). Before washing, VEERALIN® and MAGNet® LLINs induced similar level of exiting (55%) but after washing exiting was significantly greater for VEERALIN® LN (64.7%) than for MAGNet® LN (46.8%) (GLMMs, *P* < 0.0001).

**Fig. 1:**
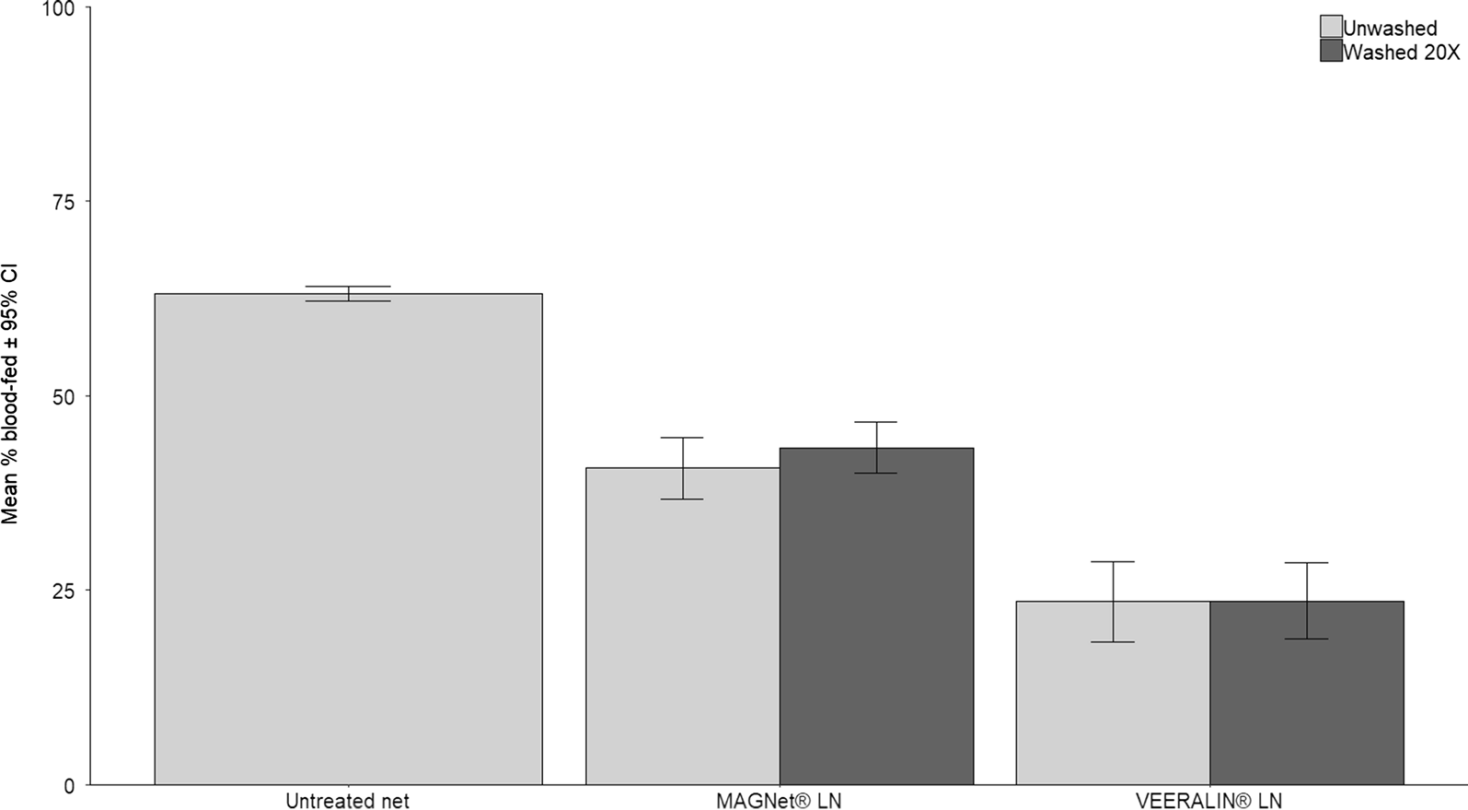
Blood-feeding rates of wild pyrethroid resistant *An. gambiae* s.s. in experimental huts in M’bé, Côte d’Ivoire. Error bars represent 95% CIs

Blood-feeding was significantly inhibited by insecticide-treated net treatment compared to the untreated control net (GLMMs, *P* < 0.0001) (Additional file 1: Table S1, Additional file 2: Table S2). Unwashed VEERALIN® LN induced significantly greater blood-feeding inhibition (62.7%) than MAGNet® LN (35.5%) (GLMMs, *P* < 0.0001) (Table 1, Fig. 1). The difference persisted after washing, being no loss of protection with either LN.

**Fig. 2:**
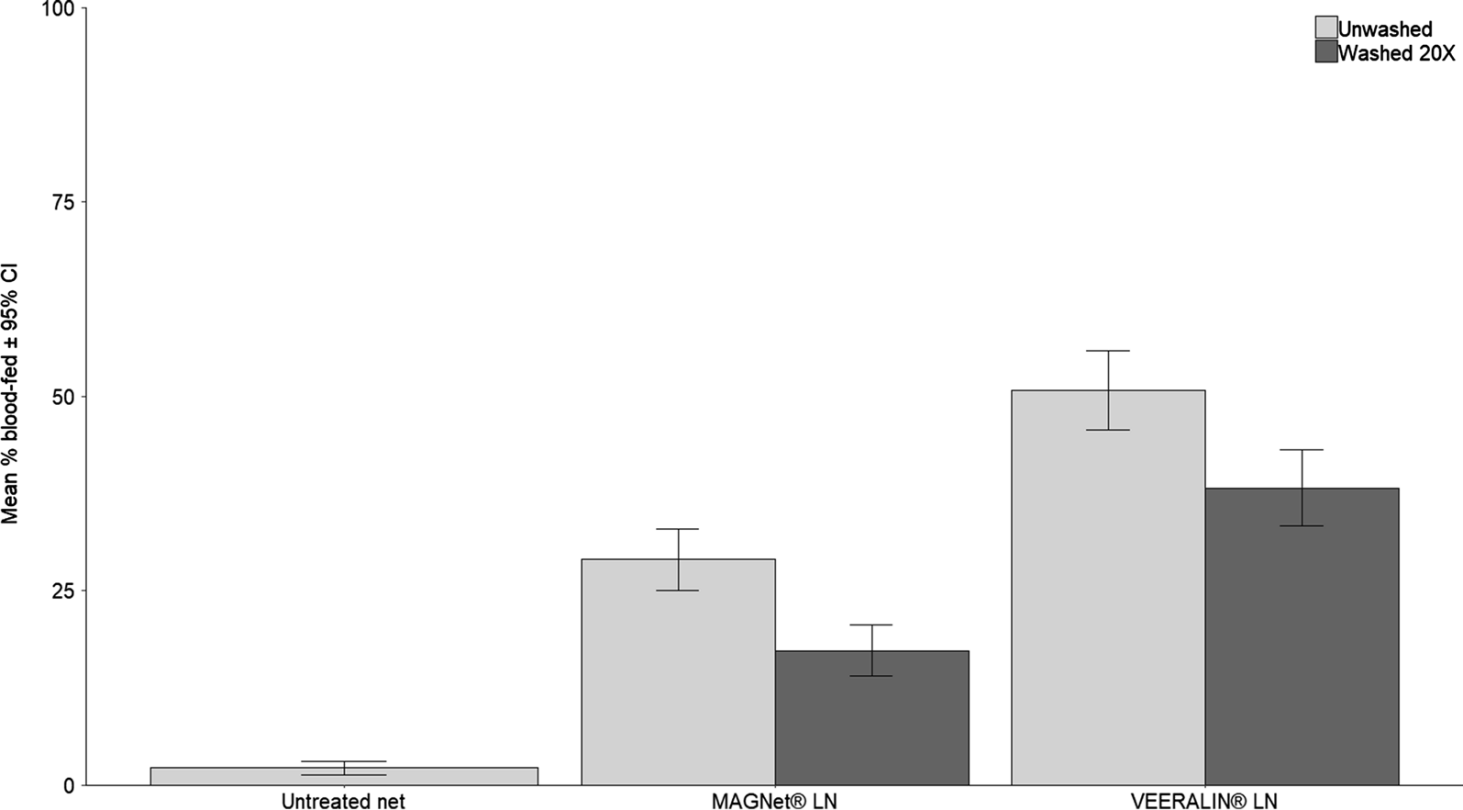
Mortality rates of wild pyrethroid resistant *An. gambiae* s.s. in experimental huts in M’bé, Côte d’Ivoire. Error bars represent 95% CIs

All insecticide-treated nets induced greater mortality than the untreated net (GLMMs, *P* < 0.0001) (Fig. 2, Additional file 1: Table S1, Additional file 2: Table S2). The unwashed VEERALIN® LN produced mortality of 51%, although this was significantly greater than that induced by MAGNet® LN unwashed (29%) (GLMMs, *P* < 0.0001). After washing, mortality with the PBO-LLIN and pyrethroid-only LLIN decreased significantly to 38.2% for VEERALIN® LN and to 17.3% for MAGNet® LN (GLMMs, *P* < 0.0001); the decrease relative to the unwashed net was 24.8% for VEERALIN® LN and 40% for MAGNet® LN.

The level of personal protection derived from the use of VEERALIN® and MAGNet® LLINs (unwashed and washed) against *An. gambiae-*biting ranged between 86.6–87.1% for VEERALIN® LN and 66.2–69% for MAGNet® LN before and after washing. The Overall Killing Effect was low (< 16%) across all treatments (Table 1). Before washing, VEERALIN® LN induced significantly greater overall killing effect (15.5%) compared to MAGNet® LN (11.8%), but the difference was not significant (GLM, *P* = 0.41). Although there was a reduction in killing effect with VEERALIN® (11.5%) and MAGNet® (6.4%) LLINs after washing, the decrease in effect was only significant with MAGNet® LN (GLM, *P* = 0.014).

### Cone bioassays

Mortality rates of the susceptible *An. gambiae* were 100% with all treated nets assayed in WHO cone at the three time points (before, after washing and after field trial).

### Chemical analysis

The mean alpha-cypermethrin content in MAGNet® and VEERALIN® LLINs and the concentration of the synergist PBO in VEERALIN® LN are presented in Tables 2 and 3. The initial concentrations of alpha-cypermethrin in VEERALIN® LN (6.91 and 6.75 g/kg) and MAGNet® LN (6.39 and 5.95 g/kg) were close to the target dose of 6 g/kg ± 25% for VEERALIN® LN and 5.8 g/kg ± 25% for MAGNet® LN, with a within-net variation of less than 10%. After washing, the alpha-cypermethrin content was 6.03 g AI/kg for VEERALIN® LN and 5.65 g AI/kg for MAGNet® LN corresponding to an overall wash retention rate of 89% for VEERALIN® LN and 95% for MAGNet® LN. After the 5-week hut trial, there was marginal decline in alpha-cypermethrin content (< 15%) with either LLIN washed or unwashed. The initial concentration of PBO in the unwashed VEERALIN® LN (2.63 g/kg) was within the acceptable range of the target dose of 2.2 g/kg ± 25% but was slightly overdosed in the VEERALIN® LN that was destined to be washed 20 times (2.95 g/kg) (Table 3). After 20 washes, there was a decrease in PBO content from 2.95 to 2.64 g AI/kg, corresponding to an overall wash retention of 89%. After hut trial, there was a small decrease in PBO content (< 20%, Table 3).

**Table 2:** Content of alpha-cypermethrin in LLINs used in the experimental hut trial

Treatment	Concentration of alpha-cypermethrin (g/kg)		
	Before trial	After washing	After trial
MAGNet® LN unwashed	6.39	–	6.47
MAGNet® LN 20 washes	5.95	5.65	5.84
VEERALIN® LN unwashed	6.91	–	7.40
VEERALIN® LN 20 washes	6.75	6.03	5.78

**Table 3:** Content of piperonyl butoxide (PBO) in VEERALIN® LN used in hut trial

Treatment	Concentration of PBO (g/kg)		
	Before trial	After washing	After trial
VEERALIN® LN unwashed	2.63	–	3.90
VEERALIN® LN 20 washes	2.95	2.64	2.40

The decrease in insecticide content after washing of VEERALIN® and MAGNet® LLINs was associated with a significant decrease in hut mortality; however, personal protection was maintained and blood-feeding rates did not differ between unwashed and 20 times washed LLINs (Tables 2, 3, Figs. 1, 2).

## Discussion

Malaria control and pyrethroid-only nets are under threat from the increasing prevalence and intensity of pyrethroid resistance among malaria vectors [34]. To preserve insecticide mosquito net technology, the most widely used form of vector-control method, and continue progress toward elimination, a class of mosquito net incorporating the synergist piperonyl butoxide (PBO) has been developed to neutralise some forms of metabolic resistance to pyrethroids. On the basis of a cluster randomised trial of Olyset® Plus LN, which demonstrated epidemiological evidence of the greater effectiveness of pyrethroid-PBO nets in areas of resistance, the WHO has conditionally endorsed pyrethroid-PBO nets as a new product class for malaria control in areas where resistance is conferred by monooxygenase-based resistance mechanisms. Apart from Olyset® Plus LN, there are several brands of PBO LLINs, which are being developed for approval by the WHO prequalification team. The purpose of the present study was to evaluate in experimental huts the efficacy of the pyrethroid-PBO net, VEERALIN® LN *versus* the pyrethroid-only net, MAGNet® LN, against pyrethroid-resistant populations of *An. gambiae* s.s. mosquitoes at the M’bé field station in Côte d’Ivoire.

In experimental huts, MAGNet® LN, an alpha-cypermethrin treated net reduced mosquito survival and blood-feeding by approximately 30% for both outcomes. This low effect size achieved by MAGNet® LN against pyrethroid-resistant *An. gambiae* s.s. mosquitoes is consistent with findings from previous experimental hut trials with pyrethroid-only LLINs performed at the same site [9, 26] and elsewhere [7, 8, 35]. This provides further evidence of the poor performance of pyrethroid LLIN in areas where malaria-vectors have developed multiple mechanisms of pyrethroid resistance [7, 36].

The addition of PBO to alpha-cypermethrin in the net was associated with a significant improvement in control and protection against mosquito bites. VEERALIN® LN killed significantly higher proportions (38–51%) of the highly resistant population of *An. gambiae* s.s. compared to MAGNet® LN (17–29%). In previous hut trials comparing pyrethroid-PBO net with pyrethroid-only nets, e.g. Olyset® Plus *versus* Olyset® LLINs or PermaNet® 3.0 *versus* PermaNet® 2.0 LLINs, the difference in induced mortality between PBO and standard LLIN could not be attributable to PBO conclusively because the original concentration of pyrethroid or the bleed rate of pyrethroid in the pyrethroid-PBO net differed from that in the pyrethroid-only LLIN [16–18]. In the present study, the loading dose of alpha-cypermethrin in VEERALIN® and MAGNet® LLINs were similar (6 and 5.8 g/kg, respectively) as was the wash retention of alpha-cypermethrin over 20 washes (89 and 95%, respectively). Therefore, the substantial increase in mortality observed with VEERALIN® LN was most likely due to the PBO component, which is known to inhibit the activity of key pyrethroid-detoxifying enzymes [10]. However, it should be noted that full control of pyrethroid-resistant mosquitoes was not achieved with VEERALIN® LN in experimental huts. This could be due to the presence of resistance mechanisms unaffected by the synergist PBO. Another plausible explanation could be that the dose of PBO (target dose of 2.2 g/kg) deployed in VEERALIN® LN and the bleed rate of PBO to the net surface (wash retention index = 98.9% per wash) was insufficient to inhibit the range of P450 enzymes associated with resistance in the local *An. gambiae* s.s. For example, in an area of Benin with increased oxidase activity, Olyset® Plus LN containing 5 times higher the loading concentration of PBO (10 g/kg) and a much higher bleed rate (wash retention index of 96% per wash) produced significantly higher mortality of the local *An. gambiae-*mosquitoes (67–81%) [18] compared to the effect size with VEERALIN® LN in the present study. Of course, the resistance situation in Benin [37] would not be directly comparable with the resistance situation in Côte d’Ivoire [24] and care should be taken not to overinterpret or compare trial data taken from different locations or times. Nevertheless, there is a significant variation in the loading dose and wash retention index of PBO in the current brands of pyrethroid-PBO nets pre-qualified by WHO. There is an urgent need for comparative trials of the different brands of pyrethroid-PBO LLINs in the same location and time in order to rank their efficacy or equivalence. The doses applied to the different brands should be informed by calibration studies designed to determine the dose and the optimal bleed rate of PBO required to fully inhibit oxidase-based resistance mechanisms in the target vector species.

Apart from the greater killing effect observed with VEERALIN® LN, there was a significant reduction in human-vector contact resulting from the high blood-feeding inhibition (60%), deterrence (> 64%), exiting of mosquitoes (55–64%) and personal protection (87%). The blood-feeding inhibition and personal protection against mosquito bites is arguably a more important attribute of pyrethroid-PBO LLIN than mortality. While the level of protection induced by VEERALIN® LN did not decrease with washing, there was a significant decrease in mortality after 20 standardized washes. Nevertheless, VEERALIN® LN remained superior in terms of mortality to MAGNet® LN washed to some extent. The significant loss in mortality and maintenance of personal protection observed with VEERALIN® LN after washing stresses the need for evaluating the durability of PBO net under operational household conditions. Reduction in mosquito mortality occurring after washing is a shortcoming common to all existing pyrethroid PBO nets. Hut trials with PermaNet® 3.0 LN performed in pyrethroid-resistant areas in Benin [15] and Côte d’Ivoire [17] showed a significant decrease in efficacy after washing both in terms of mortality and blood-feeding inhibition with the PBO net performing no better than the pyrethroid-only LLIN. A typical example is the community trial of Olyset® Plus LN in Tanzania: the PBO content under rural condition of use decreased by 83% compared to a decrease by only 42% for permethrin after 21 months. Despite this decrease in PBO content over this period, a 33% reduction in malaria-infection prevalence in children protected with Olyset® Plus LN was still observed compared to those living in area covered with Olyset® LN. The superior performance of the PBO net Olyset® Plus LN was sustained over 21 months of use in the Tanzanian study and efficacy is still being monitored to determine whether this effect is maintained over the assumed net lifespan of three years [21].

Most hut trials evaluating the efficacy of PBO nets were conducted in areas where *An. gambiae* s.s. is the predominant malaria-vector species [15, 17–19]. Hut efficacy data of PBO nets against other major malaria vectors including *An. funestus* and *An. arabiensis* is mainly confined to East Africa. In a recent WHOPES-commissioned hut trial carried out in Ifakara, Tanzania, VEERALIN® LN produced low mortality of *An. arabiensis* and *An. funestus*, which was not significantly different to MAGNet® LN [38]. This contrasts with findings from the present study and the difference in performance of VEERALIN® LN in both countries could be attributed to the inherent differences in behaviour between mosquito vector species, in the strength/mechanisms of resistance or to differences in hut design used [27].

Although the present study demonstrated the potential of VEERALIN® LN to enhance control and reduce transmission in areas compromised by pyrethroid resistance, proof of impact on malaria metrics would ideally require large scale cluster randomized trials in a West African setting. VEERALIN® LN belongs to the same class of net as Olyset® Plus LN. According to the latest WHO recommendation on deployment of PBO nets, a candidate PBO net belonging to the same class of a net for which epidemiological data are available does not need to be subjected to another CRT [39]. Instead, the effectiveness of the candidate PBO net is to be assessed using appropriate and relevant entomological endpoints as recently set forth by WHO [40]. Following the demonstration by the CRT in Muleba, Tanzania, of the benefit of PBO net over standard pyrethroid net on malaria metrics, all currently available PBO nets, have been endorsed by WHO [39]. Deployment of PBO net by National Malaria Control Programmes is now advocated for by WHO in areas where resistance is mostly driven by monooxygenase-based mechanisms. A second CRT currently underway in Uganda is evaluating two types of pyrethroid-PBO net (PermaNet® 3.0 and Olyset® Plus LLINs) [41]. This trial may provide evidence on whether the difference in dose and location of PBO between these nets under evaluation make any difference to the size of the effect on transmission. Given the recommendation to endemic countries to deploy PBO-based LLIN, it will be necessary to demonstrate that each type of pyrethroid PBO nets is efficacious against metabolic resistant *Anopheles* mosquitoes. WHO now requires that all second-in-class products need to demonstrate equivalence to the first-in-class in experimental hut conditions [39, 40]. Studies based on non-inferiority in experimental hut trials that will generate evidence on the relative entomological efficacy of all five pyrethroid PBO nets are essential to generate that knowledge and ensure impact.

## Conclusions

The pyrethroid-PBO VEERALIN® LN was more efficacious than standard pyrethroid-only MAGNet® LN in experimental huts both in terms of mosquito mortality and protection against mosquito bites and therefore meets WHO interim approval. The study provides evidence on the potential of PBO nets to enhance control of pyrethroid-resistant *An. gambiae* s.s. mosquitoes and reduce transmission in West Africa.

## Supplementary information

**Additional file 1: Table S1.** Fixed effects in generalised linear mixed effect model to evaluate the efficacy of unwashed and 20-times washed pyrethroid-PBO and pyrethroid-only LLIN against *Anopheles gambiae* s.s. mosquitoes in MʼBe, Côte d’Ivoire.

**Additional file 2: Table S2.** Random effects in generalised linear mixed effect model to evaluate the efficacy of unwashed and 20-times washed pyrethroid-PBO and pyrethroid-only LLIN against *Anopheles gambiae* s.s. mosquitoes in M’Be, Côte d’Ivoire.

## Data Availability

All data generated or analysed during this study are included in this published article.
